# Supplementary material for: Mid-Phase Hyperfluorescent Plaques Seen on Indocyanine Green Angiography in Patients with Central Serous Chorioretinopathy
Source: J Clin Med. 2021 Sep 30;10(19):4525. doi: 10.3390/jcm10194525 (PMC8509799; doi:10.3390/jcm10194525)
Supplement: Supplementary file 1 [file jcm-10-04525-s001.zip › jcm-1364341-supplementary.pdf]

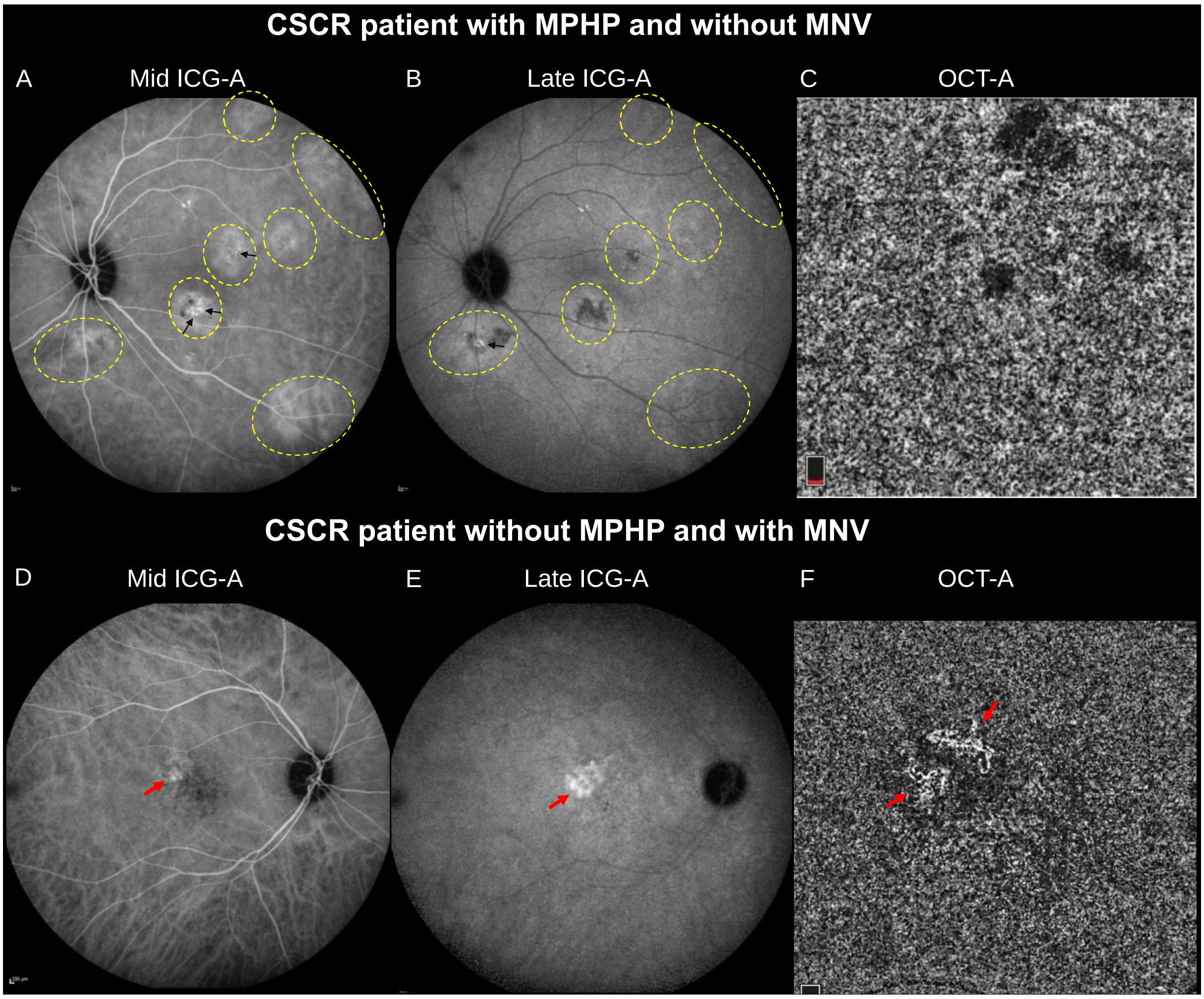

Supplementary Figure S1. A-C: Indocyanine angiography (ICG-A) and OCT-angiography (OCT-A) of a patient with central serous chorioretinopathy (CSCR). A-B: ICG-A shows mid-phase hyperfluorescent plaques (MPHP) that become isofluorescent during late-phase associated with hypofluorescent spots in around two-third of plaques. C: OCT-A does not show macular neovascularization (MNV). D-F: ICG-A and OCT-A of a CSCR patient associated with macular neovascularization (MNV). D-E: ICG-A shows a macular hyperfluorescent area (arrow, D) during mid-phase that increases in size and intensity during late phase (arrow, E). F: OCT-A shows macular neovascularization (arrow).

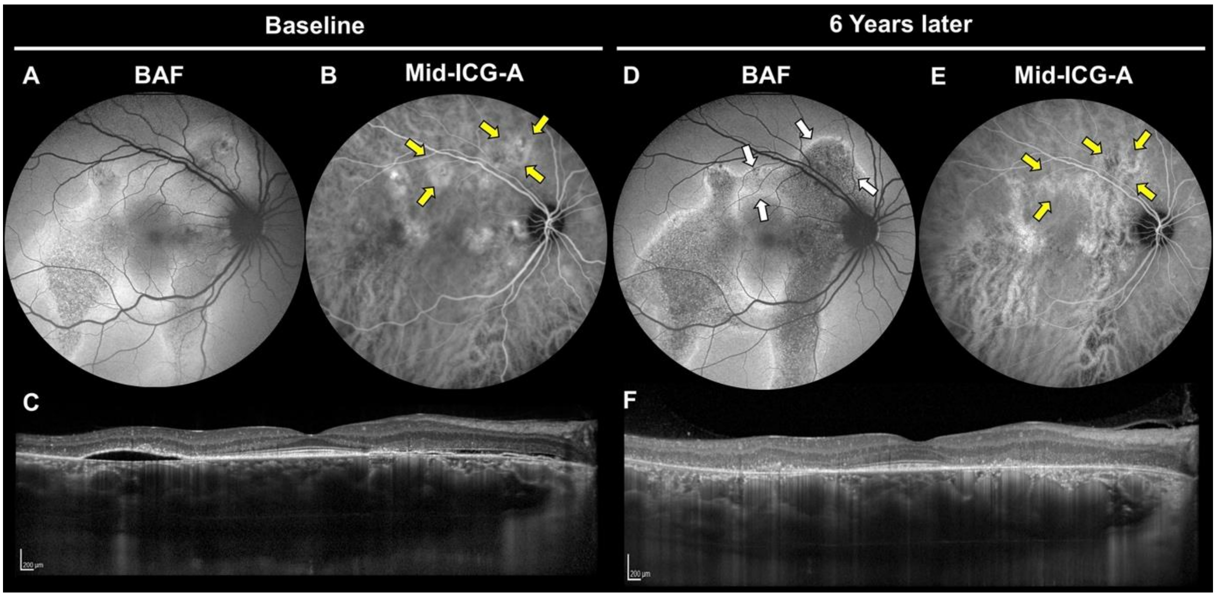

Supplementary Figure S2. Multimodal imaging of a 48-year-old man with a chronic central serous chorioretinopathy at baseline and after 6 years of follow-up. A-C: At baseline. A. Blue-light fundus autofluorescence (BAF) shows a diffuse retinal pigment epithelium (RPE) with gravitational tracks. B. Mid-phase indocyanine green angiography (ICG-A) shows multifocal mid-phase hyperfluorescent plaques (MPHP). C. The horizontal enhanced depth imaging (EDI) OCT centered on the fovea shows a thick choroid, a dome-shaped pigment epithelium detachment (PED) and an irregular PED associated with a decreased thickness of the photoreceptor layer. D-F: After 6 years of follow-up. D. BAF shows an increased area of hypo-autofluorescence (arrows). E. Mid-phase ICG-A shows that the previous MPHP area (B) are no longer visualized in the hypo-autofluorescent area (arrows). F. EDI-OCT passing through the fovea shows the resolution of the previous PED (C) with increased atrophy of the RPE and photoreceptor layer.

`
